# The Cryptococcus neoformans Flc1 Homologue Controls Calcium Homeostasis and Confers Fungal Pathogenicity in the Infected Hosts

**DOI:** 10.1128/mbio.02253-22

**Published:** 2022-09-28

**Authors:** Piotr R. Stempinski, Kristie D. Goughenour, Lukas M. du Plooy, J. Andrew Alspaugh, Michal A. Olszewski, Lukasz Kozubowski

**Affiliations:** a Department of Genetics and Biochemistry, Eukaryotic Pathogens Innovation Center (EPIC), Clemson University, Clemson, South Carolina, USA; b LTC Charles S. Kettles VA Medical Center, Ann Arbor, Michigan, USA; c Division of Pulmonary and Critical Care Medicine, Department of Internal Medicine, University of Michigan Medical School; d Departments of Medicine and Molecular Genetics/Microbiology, Duke University Medical Center, Durham, North Carolina, USA; University of Minnesota Medical School

**Keywords:** *Cryptococcus neoformans*, autophagy, calcium signaling, opportunistic fungi, pathogenesis

## Abstract

Cryptococcus neoformans, an opportunistic yeast pathogen, relies on a complex network of stress response pathways that allow for proliferation in the host. In Saccharomyces cerevisiae, stress responses are regulated by integral membrane proteins containing a transient receptor potential (TRP) domain, including the flavin carrier protein 1 (Flc1), which regulates calcium homeostasis and flavin transport. Here, we report that deletion of C. neoformans *FLC1* results in cytosolic calcium elevation and increased nuclear content of calcineurin-dependent transcription factor Crz1, which is associated with an aberrant cell wall chitin overaccumulation observed in the *flc1*Δ mutant. Absence of Flc1 or inhibition of calcineurin with cyclosporine A prevents vacuolar fusion under conditions of combined osmotic and temperature stress, which is reversed in the *flc1*Δ mutant by the inhibition of TORC1 kinase with rapamycin. Flc1-deficient yeasts exhibit compromised vacuolar fusion under starvation conditions, including conditions that stimulate formation of carbohydrate capsule. Consequently, the *flc1*Δ mutant fails to proliferate under low nutrient conditions and displays a defect in capsule formation. Consistent with the previously uncharacterized role of Flc1 in vacuolar biogenesis, we find that Flc1 localizes to the vacuole. The *flc1*Δ mutant presents a survival defect in J774A.1 macrophage cell-line and profound virulence attenuation in both the Galleria mellonella and mouse pulmonary infection models, demonstrating that Flc1 is essential for pathogenicity. Thus, cryptococcal Flc1 functions in calcium homeostasis and links calcineurin and TOR signaling with vacuolar biogenesis to promote survival under conditions associated with vacuolar fusion required for this pathogen’s fitness and virulence.

## INTRODUCTION

Cryptococcus neoformans is pathogenic yeast, which mostly causes disease in individuals with compromised immune system (1, 2). Cryptococcosis is initiated by the inhalation of spores or yeast cells and then develops into a pulmonary infection, which can disseminate through blood vessels to the central nervous system and cause cryptococcal meningoencephalitis (3, 4). Survival in the human host requires the ability of this pathogen to adjust to elevated mammalian body temperature, low nutrients, and osmotic/oxidative stress (5, 6). The success of C. neoformans pathogenesis is attributed to a number of features, including an ability to proliferate at 37°C, formation of polysaccharide capsule, melanization, and morphological transition into polyploid titan cells (7).

While several pathways responsible for the cryptococcal fitness and ability to withstand the pressure of the immune system have been studied (6, 8–10), little is known about the role of cryptococcal transient receptor potential channels (TRP channels). These ion channels with six membrane-spanning helices modulate Ca^2+^ transport and contribute to the regulation of multiple intracellular processes in mammalian cells (11, 12). For example, human Mucolipin 1 (TRPML1/MCOLN1) is a TRP channel acting in TORC1 and calmodulin-dependent pathways that regulate vacuolar fusion, membrane trafficking, exocytosis, and autophagy (13–15).

In fungal cells, proteins that contain TRP domains belong to the fungal-specific FLC family (16). Fungal FLC’s play an important role in calcium homeostasis, cell growth regulation, cell wall integrity, response to osmotic shock, and flavin transport (17–22). The Flc1 homologue was previously characterized in *C. deneoformans* (form. C. neoformans var*. neoformans*) as a potential flavin carrier: a homologue of four genes in the genome of S. cerevisiae encoding putative flavin carrier proteins (*Flc1*, *Flc2*, *Flc3*, *YOR365C*) (23). However, a recent study in S. cerevisiae suggested the involvement of Flc1 homologues in calcium signaling (17–19, 21, 22, 24). Additionally, the *PKD2* gene encoding a TRP-like protein in S. pombe is essential, and double deletion of *FLC1* and *FLC2* in S. cerevisiae is synthetically lethal (17, 18). In contrast, elimination of *FLC1* in *C. deneoformans* does not compromise growth under nonstress conditions, but limits its growth in nutrient-depleted and high temperature conditions (23). The role of Flc1 in the most important pathogen of the Cryptococcus genus, C. neoformans (formerly C. neoformans var. *grubii*), has not been studied. Given its potential importance in Ca^2+^ homeostasis, which is then crucial for cryptococcal thermotolerance and virulence (25–27), we tested the hypothesis that Flc1 contributes to C. neoformans fitness and persistence during infection.

Here, we report the crucial role of Flc1 for C. neoformans stress responses and virulence, which is linked to its role in Ca^2^**^+^** signaling/homeostasis. Our novel data demonstrate that Flc1 deficiency in C. neoformans results in abnormal distribution of chitin, inhibition of the vacuolar fusion, and interruption of autophagy. Importantly, cells lacking *FLC1* display a significant loss of virulence in both the invertebrate and mammalian infection models, identifying Flc1 protein as a promising potential drug target.

## RESULTS

### *In silico* analysis of the C. neoformans Flc1 homologue.

**FIG 1:**
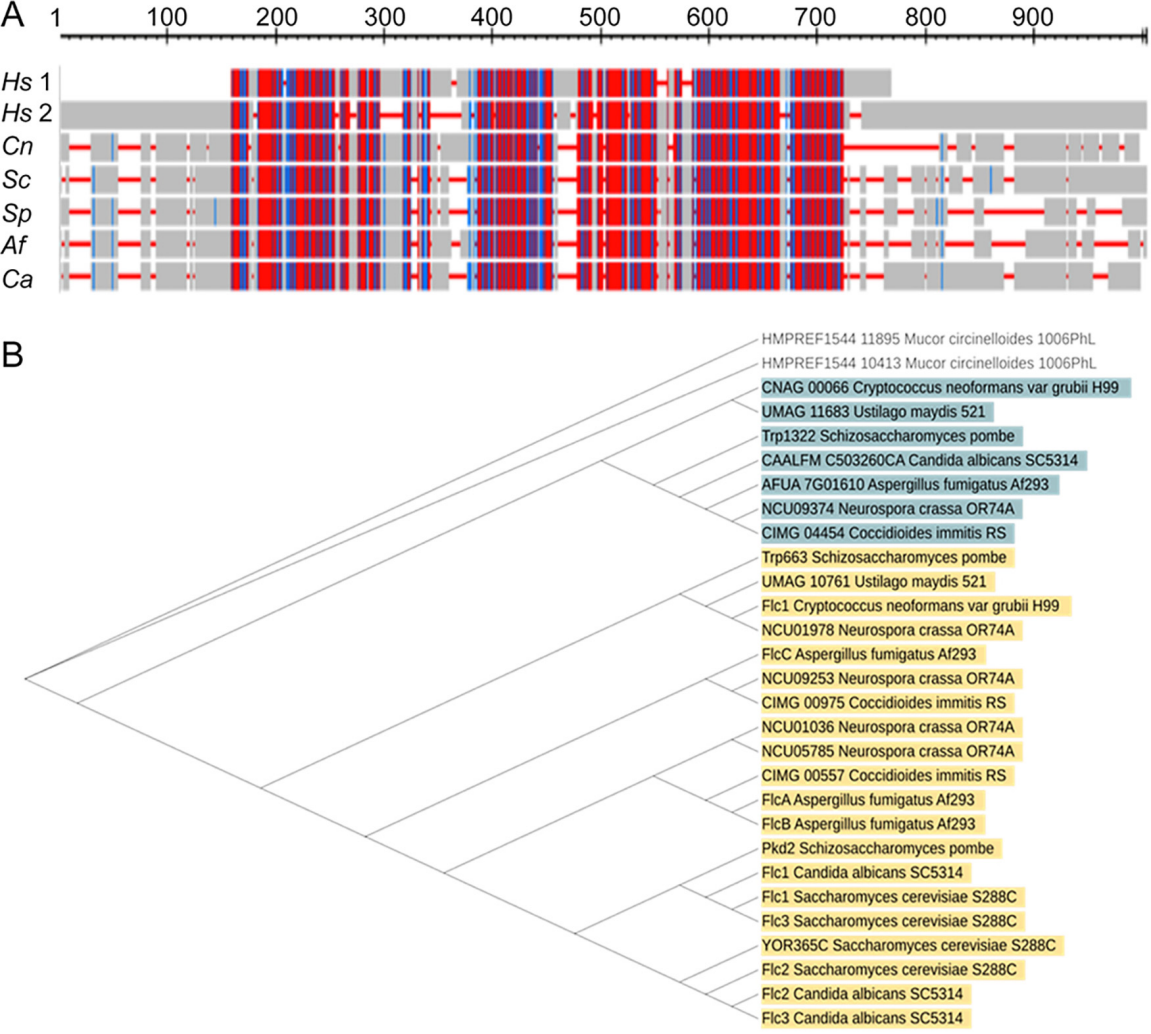
*In silico* analysis of the Flc1 sequence. (A) Graphical representation of multiple sequence alignment, performed with COBALT software, for human Mukolipin-1 (*Hs* 1), Policystin-2 (*Hs* 2), and fungal homologues of Flc1 including Cryptococcus neoformans (*Cn*), Saccharomyces cerevisiae (*Sc*), Schizosaccharomyces pombe (*Sp*), Aspergillus fumigatus (*Af*), and Candida albicans (*Ca*). This method highlights conserved, and less conserved sequences based on relative entropy threshold for each residue. More conserved sequences are highlighted in red and less conserved sequences are highlighted in blue. (B) Phylogenetic tree of TRP domain proteins from fungal species that belong to Ascomycota, Basidiomycota, and Mucormycota. Phylogenetic analysis was performed using the neighbor-joining method. Results of the protein homology analysis for the proteins selected for phylogenetic analysis are presented in the Table S1.

While the Flc1 homologue has been identified in *C. denoformans* (23), this potential drug target has not been studied in the most clinically prevalent species C. neoformans. *In silico* analysis revealed that the C. neoformans CNAG_04283 gene codes for a Flc1 homologue, containing 766 amino acids (aa) and two domain regions: a mucolipin-like (ML) TRP_N domain (PF14558) and TRP domain (PF06011). According to BLAST search for protein sequence homology, C. neoformans Flc1 shows considerable level of aa identity (22 to 29%) with FLC homologues in model yeasts and clinically important pathogenic fungi, Aspergillus fumigatus, Candida albicans, and Mucor circinelloides (Fig. 1; Table S1). Importantly, while these fungal FLC proteins share predicted TRP domains with human TRP channels, Mucolipin 1 (TRPML1) and Polycystic Kidney Disease 2 protein (Pkd2), homology analysis with EMBOSS Needle reveals a very limited aa conservation with human proteins (aa identity between C. neoformans Flc1 and TRPML1 and Pkd2 is 1.1 and 2.4%, respectively).

10.1128/mbio.02253-22.2 TABLE S1 Proteins with significant homology (% identity is indicated) to C. neoformans Flc1. Download Table S1, PDF file, 0.1 MB. Copyright © 2022 Stempinski et al. 2022 Stempinski et al. https://creativecommons.org/licenses/by/4.0/ This content is distributed under the terms of the Creative Commons Attribution 4.0 International license.

Comparative analysis of C. neoformans Flc1 with proteins from other fungal species revealed the existence of multiple FLC-like proteins containing a TRP domain (PF06011). We found at least two FLC-like homologues in each of the analyzed fungal species, indicating its importance and conserved role in fungi (Table S1). All homologues of Flc1 contain TRP domains (PF06011), and most of the tested Flc1 homologues from the phyla Ascomycota, Basidiomycota, and Entomophthoromycota contain an ML-like (PF14558) or ML (PF02221) domain (in the case of Mucormycota).

Phylogenetic analysis of FLC-like proteins in ascomycetous and basidiomycetous species revealed the existence of two subgroups of FLC proteins (Fig. 1). In ascomycetous fungi, FLC homologues that belong to the subgroup A (highlighted in yellow in Fig. 1B) are represented by several proteins. Interestingly, proteins that belong to the subgroup B (highlighted in blue) are represented by only one protein, have relatively longer amino acid sequences (~40% for Ascomycota and ~70% for Basidiomycota), and in most cases do not contain ML-like domain (PF14558).

### Flc1 plays a role in calcium homeostasis.

**FIG 2:**
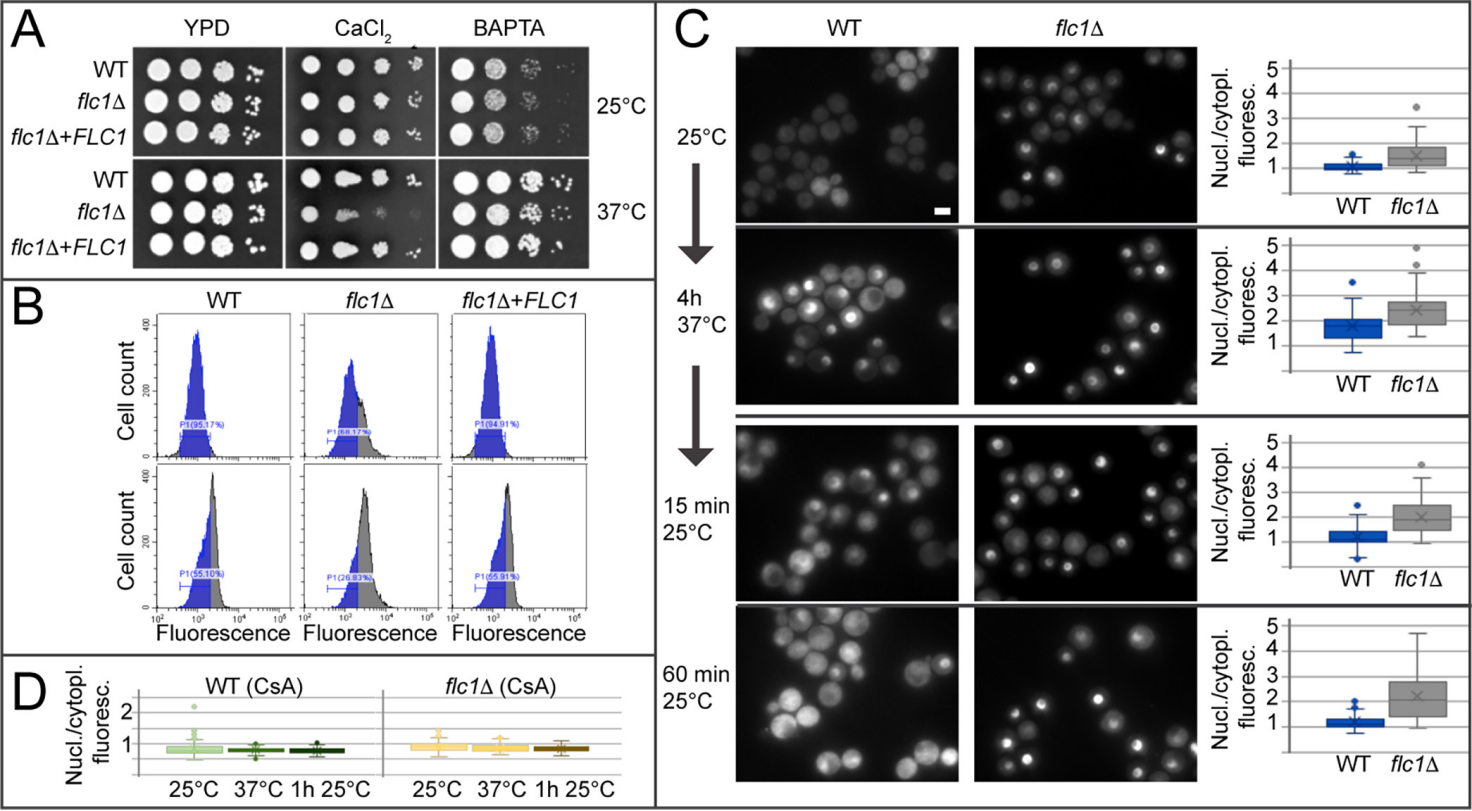
Deletion of *FLC1* impairs intracellular calcium homeostasis. (A) C. neoformans Flc1 is involved in calcium tolerance at high temperature. Ten-fold serial dilutions of cell cultures were spotted onto YPD plates containing 250 mM CaCl_2_ or 1 mM calcium chelator BAPTA. Plates were incubated for 2 days at ~25 (RT) or 37°C. (B) Deletion of *FLC1* leads to an increased level of intracellular calcium. The relative level of calcium was measured with fluorescence flow cytometry based on calcium sensitive dye Fluo-3 AM. The gray shaded area within each peak represents cells with fluorescent signal above the maximum signal detected in 95% of the WT population at RT (marked by the blue area in each peak) (C) Deletion of *FLC1* leads to increased nuclear localization of Crz1. In WT cells incubated at RT, Crz1-mCherry is not enriched in the nuclei (nuclear/cytoplasmic ratio for the Crz1-mCherry fluorescence is ~1). Incubation of the cells at 37°C promotes translocation of Crz1-mCherry to the nuclei. Nuclear and cytoplasmic fluorescence of the Crz1-mCherry begins to equalize 15 min after the transfer of WT cells from 37°C to RT, and after 60 min, Crz1-mcherry localization reverts to the normal state. The nuclear localization of Crz1-mCherry in *flc1*Δ mutant cells incubated at RT and 37°C is relatively higher than the WT. Even 60 min after the transfer from 37°C to RT, Crz1-mCherry remains enriched in the nuclei in *flc1*Δ cells. Scale bars are 5 μm. Graphs depict quantification of the ratio of nuclear/cytoplasmic fluorescence of Crz1-mCherry (*n* > 90). (D) Changes in Crz1-mCherry localization in *flc1*Δ are caused by hyper-activation of calcineurin pathway. Inhibition of calcineurin prevents high temperature induced translocation of Crz1-mCherry to the nuclei in WT and *flc1*Δ mutant cells. Quantification of the nuclear/cytoplasmic fluorescence of Crz1-mCherry was performed in cells treated with cyclosporine A (CsA; 100 μg/mL). Cultures were incubated at RT or at 37°C (*n* > 90).

*C. deneoformans* Flc1, as a homologue of four genes in the genome of S. cerevisiae encoding putative flavin carrier proteins (Flc1, Flc2, Flc3, YOR365C), was proposed to function as a flavin carrier (23). However, recent studies in S. cerevisiae suggested involvement of FLC homologues in calcium signaling (17–19, 21, 22, 24). To study the potential role of the C. neoformans Flc1 in calcium signaling, the wild type (WT), *flc1*Δ mutant, and a complemented strain (*flc1*Δ+*FLC1*) were grown on yeast extract-peptone-dextrose (YPD) medium with various levels of calcium: Ca^high^ (w/250 mM CaCl_2_) or Ca^null^ (w/BAPTA calcium chelator). Elevated external calcium suppressed cell growth of the *flc1*Δ mutant at 37°C but not room temperature (RT) (~25°C), while the presence of BAPTA did not have a significant impact on cell growth (Fig. 2A), suggesting the involvement of Flc1 in Ca-regulated high temperature stress response and predicting increased levels of the intracellular Ca^2+^ in the *flc1*Δ mutant. Consistently, flow cytometry analysis of intracellular Ca^2+^, using the fluorescent indicator Fluo-3, revealed that the *flc1*Δ mutant contained elevated Ca^2+^ signal relative to the WT and complemented strains at both RT and 37°C (Fig. 2B). Collectively, these results support a role of Flc1 in calcium homeostasis.

### Deletion of *FLC1* affects the calcineurin pathway.

Based on established C. neoformans calcium/calcineurin signaling models (28), we hypothesized that Flc1 modulates calcium-dependent functions of calcineurin. To test this, WT and *flc1*Δ mutant strains expressing the calcineurin-dependent transcription factor Crz1-mCherry were used to compare calcineurin activity based on nuclear translocation of Crz1-mCherry following calcineurin activation (29). The average ratio of the nuclear/cytoplasmic Crz1-mCherry fluorescence was approximately equal to 1 at ~25°C (RT) and increased above 1 upon incubation at 37°C for 4 h in the WT, as reported (29) (Fig. 2C). In *flc1*Δ, the relative fluorescent signal corresponding to Crz1-mCherry in the nucleus was elevated even at RT (Fig. 2C), and the ratio of the nuclear/cytoplasmic Crz1-mCherry signal at 37°C was significantly greater than that in WT. Furthermore, the Crz1-mCherry signal equalization between the nucleus and the cytoplasm following transfer from 37°C to RT, observed in the WT, was significantly delayed (over 60 min) in *flc1*Δ cells (Fig. 2C). The assay was validated using cyclosporine A (CsA), an inhibitor of calcineurin activity, with both the WT and *flc1*Δ mutant cells showing an average nuclear/cytoplasmic fluorescence ratio of ~1 at all conditions in the presence of CsA (Fig. 2D). Together, these results suggest Flc1 plays an important role in the modulation of the calcineurin-Crz1 stress response pathway in C. neoformans.

### Deletion of *FLC1* affects cell wall integrity.

**FIG 3:**
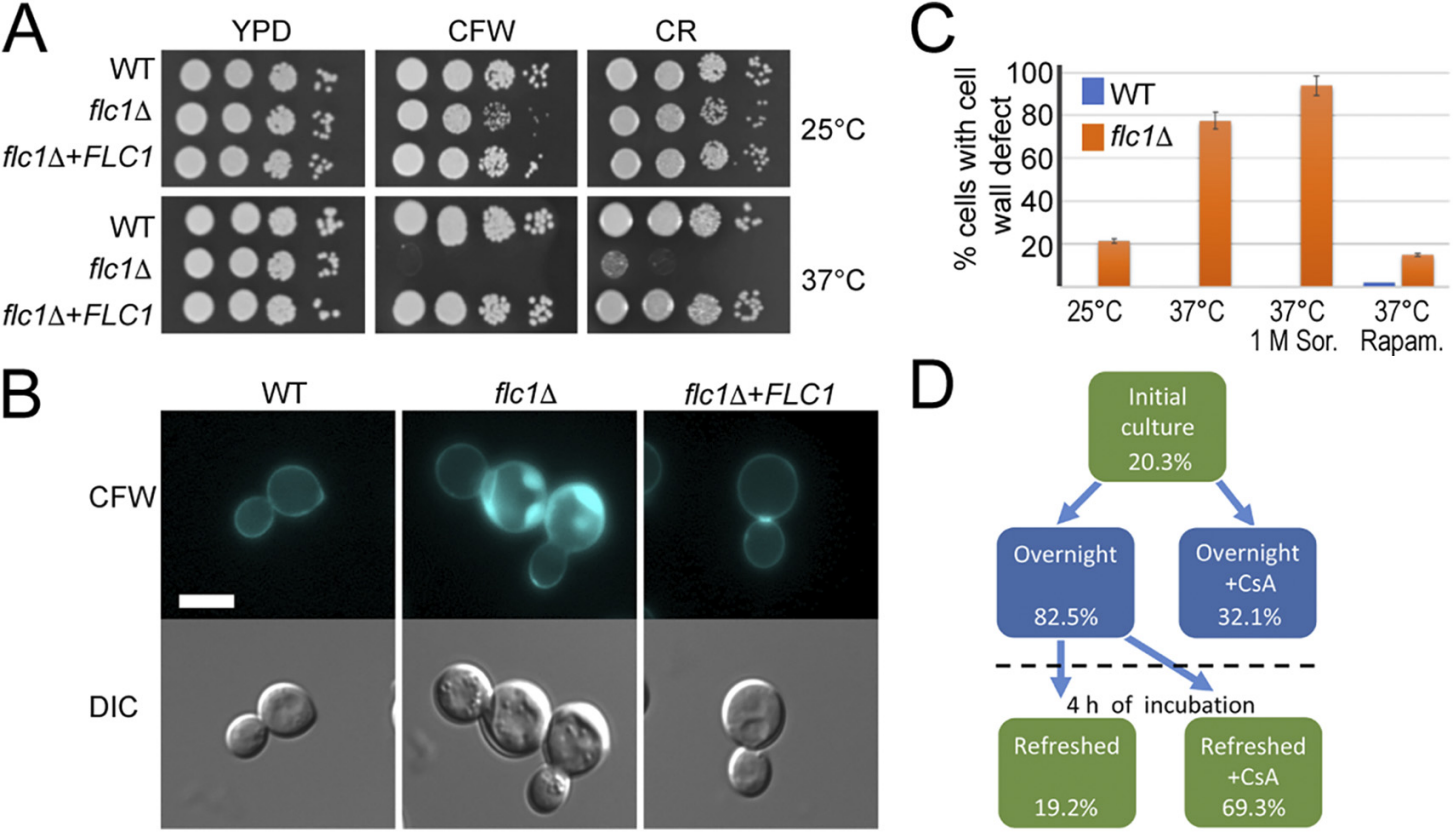
Deletion of *FLC1* has a negative effect on cell wall integrity in C. neoformans. (A) Ten-fold serial dilution of cell cultures were plated onto YPD plates containing 3.5 mg/mL Calcofluor white (CFW) or 0.8% Congo red (CR). Plates were incubated for 2 days at ~25 (RT) or 37°C. (B) Imaging of chitin overaccumulation in cells lacking Flc1. Refreshed overnight cultures were stained with CFW and examined under fluorescence microscope. Scale bar represents 5 μm. (C) Cell wall damage in *flc1*Δ cells can be stimulated by stress factors. Graph represents percentage of cell population with visible defect in chitin overaccumulation in WT and the *flc1*Δ mutant. Cells were incubated at various conditions (RT, 37°C, 37°C with 1 M sorbitol, and 37°C with 1 ng/mL rapamycin) for 18 h and refreshed for 4 h before staining with CFW and microscopic observation. Experiment was performed in three biological replicates. (D) Overaccumulation of chitin spots in *flc1*Δ cells correlates with stationary-phase of cell culture growth and is dependent on calcineurin activity. Microscopic analysis of *flc1*Δ cells stained with CFW indicated increased number of cells with cell wall defect among cells in stationary-phase of growth. Treatment of cell culture with the inhibitor of calcineurin led to a reduced number of cells with overaccumulated chitin.

Given that calcineurin promotes maintenance of cell wall integrity during stress, we tested whether elimination of Flc1 affects cell wall integrity due to hyperactivation of the calcineurin pathway. Growth of the WT, the *flc1*Δ mutant, and the complemented *flc1*Δ+*FLC1* strain was compared on media supplemented with the cell wall damaging agents Calcofluor white (CFW) and Congo red (CR). At RT, CR had no visible impact on growth of the *flc1*Δ mutant, while CFW affected it modestly (Fig. 3A). At 37°C, both CFW and CR severely affected growth of *flc1*Δ cells, consistent with Flc1’s importance in cell wall remodeling during high temperature stress.

To explore the impact of Flc1 on cell wall composition, cell wall chitin distribution was examined by staining with CFW (Fig. 3B). WT cells exhibited a uniform chitin distribution throughout the cell wall, while a significant fraction of the *flc1*Δ population presented “punctate” chitin accumulation, similar to cell wall damage reported in the *C. deneoformans flc1*Δ (23). The puncta of accumulated chitin were located at the mother-bud neck in some of the budding *flc1*Δ cells, whereas both budded and unbudded cells contained aberrant chitin puncta at random locations throughout the cell surface (Fig. 3B). Consistent with the increased sensitivity of the *flc1*Δ mutant to cell wall-damaging agents at high temperatures, the excessive chitin accumulation was more pronounced at 37°C with nearly 80% of the *flc1*Δ cells exhibiting abnormal chitin accumulation, in contrast to the ~20% observed at RT (Fig. 3C). Growth of mutants with compromised cell walls can often be rescued by osmotic stabilization of the media with 1 M sorbitol (30). Interestingly, incubation of *flc1*Δ cells at 37°C, in media supplemented with 1 M sorbitol, led to a further increase in excessive chitin accumulation, but had no effect on the WT (Fig. 3C).

The cell wall integrity pathway is subject to cross talk with other stress-related pathways, including calcineurin and TOR signaling (31). We, therefore, tested if calcineurin activity is required for chitin overaccumulation in *flc1*Δ cells. Addition of the calcineurin inhibitor CsA during overnight incubation significantly reduced the number of *flc1*Δ cells exhibiting excessive chitin accumulation (Fig. 3D). When the overnight *flc1*Δ culture, which contained ~82% of cells exhibiting excessive chitin accumulation, was subsequently refreshed with new media and grown for 4 additional hours, the percentage of cells with overaccumulated chitin was reduced to 19.4%. Interestingly, addition of CsA during the 4-h incubation after refreshing the culture had only a moderate effect, as chitin overaccumulation was still visible in ~69% of cells (Fig. 3D).

Likewise, pharmacological inhibition of the TOR pathway with rapamycin profoundly decreased the number of *flc1*Δ cells with chitin accumulation defects at 37°C (Fig. 3C), which in turn suggested a connection between Flc1 and nutrient sensing. Therefore, we compared the accumulation of chitin puncta in actively growing *flc1*Δ cells (postrefreshing the overnight culture with fresh YPD every 4 h). A total of 82.5% of the *flc1*Δ cells from stationary culture displayed chitin puncta, in contrast to only ~20% of the *flc1*Δ cells exhibiting this defect in the refreshed culture (Fig. 3D), consistent with Flc1 involvement in nutrient deprivation response.

Collectively, our data support the notion that Flc1 is involved in the response to low nutrient, osmotic, and high temperature stresses, and impacts cell wall integrity pathway, most likely through regulating intracellular calcium/calcineurin that lead to Crz1-dependent deposition of cell wall chitin.

### Deletion of *FLC1* affects localization of septins in C. neoformans.

**FIG 4:**
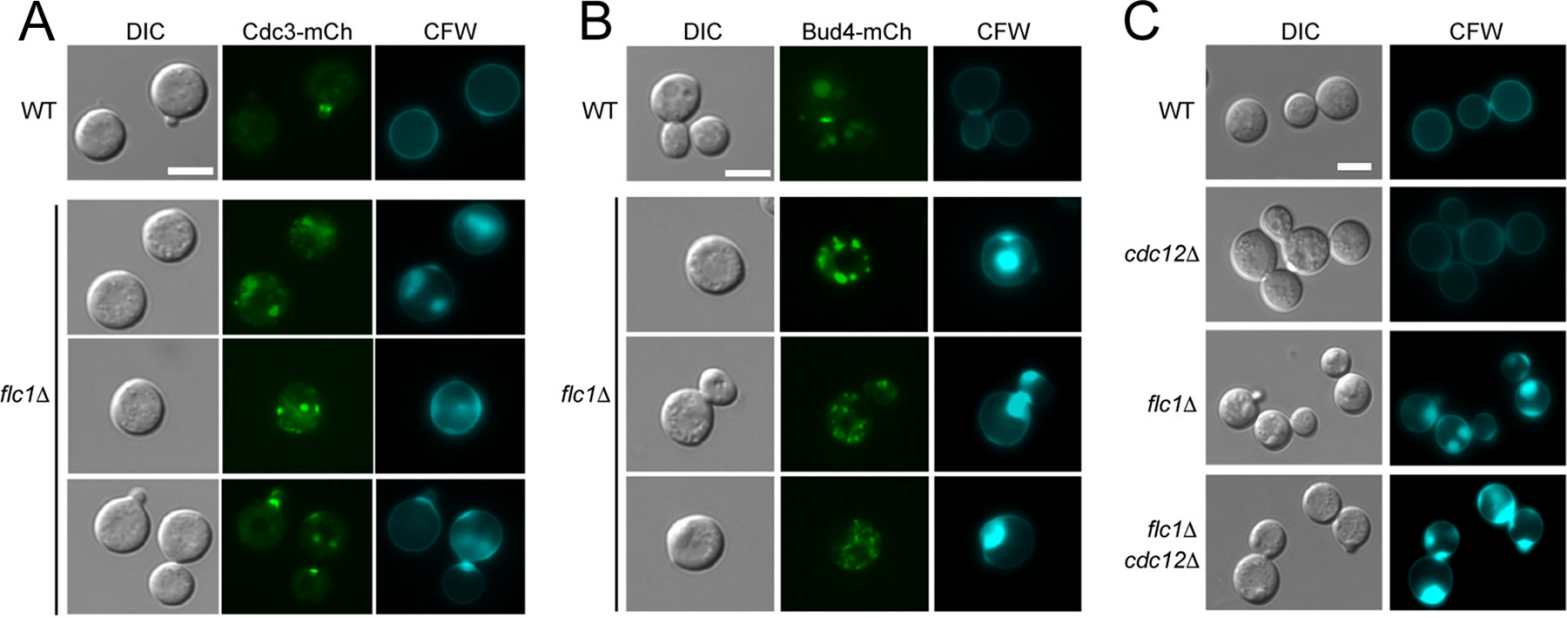
Overaccumulated chitin in *flc1*Δ cells colocalizes with septin Cdc3. Epifluorescent microscopy analysis of the *flc1*Δ mutant reveals aberrant localization of septin Cdc3-mCherry that colocalizes with abnormal chitin patches. Cells were stained with Calcofluor white (CFW). (B) Epifluorescent microscopy reveals no colocalization for Bud4-mCherry and aberrant chitin patches. Cells were stained with CFW. (C) Deletion of the gene encoding septin Cdc12 does not prevent formation of aberrant chitin patches in *flc1*Δ mutant cells. Microscopic observation of cells stained with CFW presented no visible effect on reduction of chitin patches in double deletion mutant (*flc1*Δ *cdc12*Δ). Scale bars represent 5 μm.

The septin complex plays an important role in promoting cell wall formation at the mother-bud neck in both C. neoformans and S. cerevisiae (32, 33). To explore the role of the septin complex in the overaccumulation of chitin observed in *flc1*Δ, we tagged the Cdc3 septin protein with mCherry. WT cells showed septins localized to the mother-bud neck, as expected (33). In contrast, the *flc1*Δ cells presented unusual septin localization at plasma membrane puncta (Fig. 4). These atypical punctate signals corresponding to Cdc3-mCherry colocalized with the overaccumulated chitin agglomerates visualized by CFW staining (Fig. 4). To analyze if other components of the septin complex were also localized at these aberrant chitin patches, we analyzed the localization of Bud4, an anillin-like protein that participates in septin organization during cytokinesis in S. cerevisiae (34) and likely plays a similar role in C. neoformans (35). Examination of the Bud4-mCherry localization in the *flc1*Δ cells revealed no significant colocalization with the aberrant chitin spots (Fig. 4).

Elimination of the single septin Cdc12 is sufficient to prevent formation of a fully functional septin complex (33). To test if the aberrant distribution of the septin complex is responsible for the abnormal chitin accumulation, we analyzed chitin accumulation in the *flc1*Δ *cdc12*Δ double deletion mutant. Surprisingly, elimination of Cdc12 (which predictably leads to elimination of the septin complex) did not result in the diminishment of the chitin overaccumulation in cells lacking Flc1 (Fig. 4). Thus, despite irregular septin accumulation in the *flc1*Δ cell wall, the mechanism of its aberrant chitin accumulation is independent of septin complex formation.

### Deletion of *FLC1* sensitizes the cells to the combination of osmotic and temperature shock and affects vacuolar fusion.

**FIG 5:**
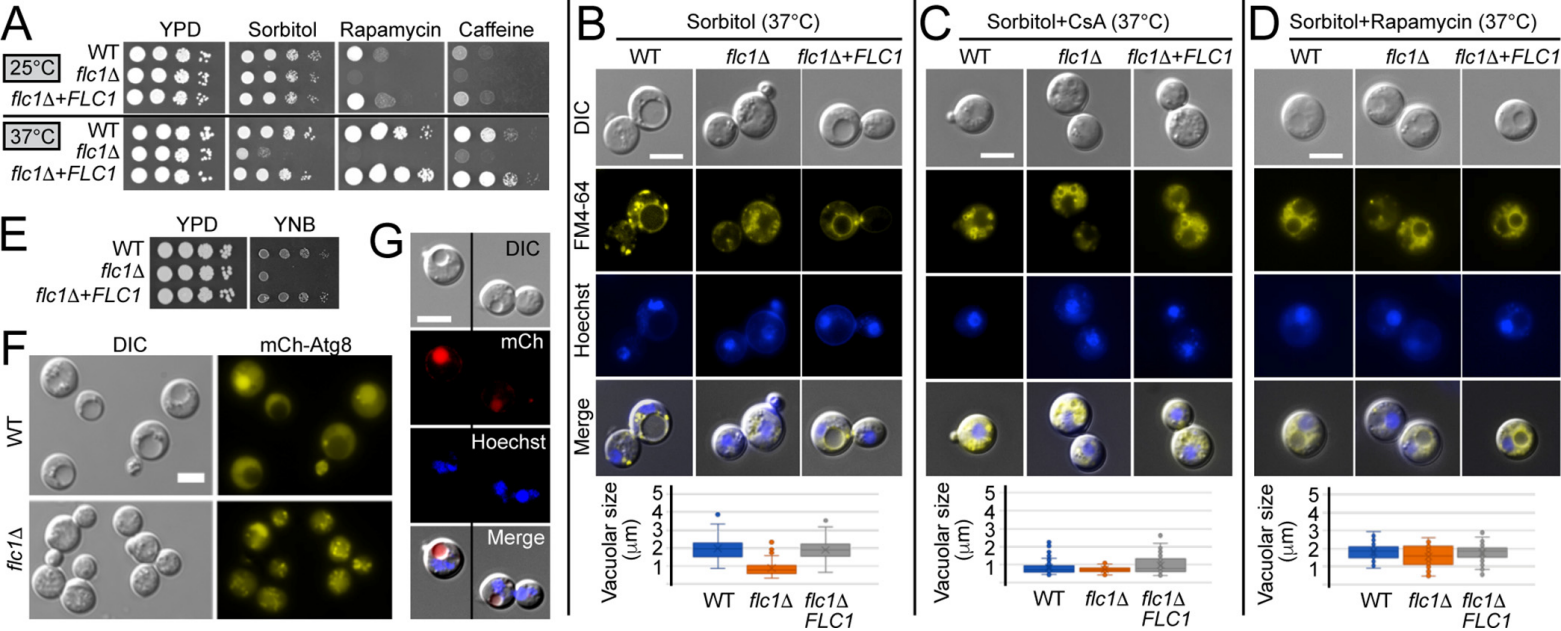
Flc1 regulates vacuolar fusion. (A) Deletion of Flc1 increases sensitivity of C. neoformans to TORC1 inhibition and combination of osmotic and thermal stress. Ten-fold serial dilutions of cells were spotted onto YPD plates containing 1 M sorbitol, or 3 ng/mL rapamycin, or 1 mg/mL caffeine and incubated for 2 to 5 days at ~25 (RT) and 37°C. (B) Analysis of vacuoles in cells exposed to thermal and osmotic shock. Cells were incubated for 4 h at 37°C in YPD media supplemented with 1 M sorbitol. Vacuoles and cell nuclei were visualized with FM4-64 and Hoechst staining, respectively. WT and the complemented strain formed large vacuoles. In contrast, formation of large vacuoles was disturbed in *flc1*Δ cells. Bar graphs represent average size of the vacuoles in the cells (*n* > 80). Average size of the vacuoles in *flc1*Δ cells differed significantly from that of the positive control (*P* < 0.001, t-test). (C) Inhibition of calcineurin with cyclosporine A (CsA) prevents formation of large vacuoles. Cells were incubated for 4 h at 37°C in YPD media supplemented with 1 M sorbitol and 100 μg/mL CsA. Bar graphs represent average size of the vacuoles in the cells (*n* > 80). (D) Inhibition of TORC1 with rapamycin restores formation of large vacuoles in the *flc1*Δ mutant. Cells were incubated for 4 h at 37°C in YPD media supplemented with 1 M sorbitol and 1 μg/mL rapamycin. Bar graphs represent average size of the vacuoles in the cells (*n* > 80). (E) Flc1 is required for growth in low nutrient condition. 10-fold serial dilutions of cells were spotted onto YPD or YNB medium and incubated at RT. (F) WT and *flc1*Δ mutant cells were incubated for 4 h in YNB media to induce process of autophagy. Observation of mCherry-Atg8 localization revealed that *flc1*Δ mutant cells display interrupted vacuolar fusion in low-nutrient conditions. (G) Flc1 localizes to the vacuoles in C. neoformans. In cells incubated at 37°C, mCherry-tagged Flc1, indicated in red, localizes to the vacuoles (visible in the DIC image). Blue color indicates nuclei stained with Hoechst. Scale bars represent 5 μm.

The exacerbated cell wall defect in the *flc1*Δ cells at 37°C in the presence of 1 M sorbitol led to a hypothesis that C. neoformans Flc1 plays a role similar to the human TRPML1. To investigate this potential functional homology, we focused on the connection with the TOR pathway and the potential role in osmotic balance (36). We found cells lacking Flc1 particularly sensitive to the combination of osmotic and thermal stress (Fig. 5A). In contrast to *C. deneoformans* (23), deletion of *FLC1* in C. neoformans significantly increased sensitivity to TOR complex 1 (TORC1) inhibition induced by rapamycin or caffeine (Fig. 5A).

To further analyze the effect of osmotic shock combined with high temperature stress, we examined vacuolar morphology. Incubation of WT cells at 37°C in the presence of 1 M sorbitol resulted in accumulation of large vacuoles, based on differential interference contrast (DIC) microscopy and the fluorescent signal of the membrane stained with FM4-64 (Fig. 5B). The majority of WT cells and the complemented mutant contained a large (>1.5 μm) vacuole. In contrast, *flc1*Δ cells contained mostly fragmented or relatively small vesicles while large vacuoles were rarely observed (Fig. 5B). To explore the potential involvement of calcineurin in vacuolar formation at 37°C/1 M sorbitol conditions, cells were incubated with addition of CsA. Inhibition of calcineurin had a visible impact on vacuolar size (Fig. 5C). The average size of vacuoles in CsA-treated WT cells was similar to the size of vacuoles in cells lacking Flc1 that were not treated with CsA (Fig. 5B and C). However, in contrast to *flc1*Δ cells that retained active calcineurin, WT cells treated with CsA contained mostly whole vacuoles of small size rather than fragmented vacuoles as frequently observed in the *flc1*Δ mutant (Fig. 5). TORC1 kinase has been shown to regulate vacuolar homeostasis in response to osmotic and low nutrient stresses (37, 38). To test if Flc1 plays a role in vacuolar morphogenesis by modulating the TOR pathway, *flc1*Δ cells were incubated with the addition of rapamycin. Addition of rapamycin significantly improved the ability of *flc1*Δ cells to form large vacuoles under osmotic stress combined with 37°C (Fig. 5D). Collectively, these data suggest that sustained activation of TORC1 in cells lacking Flc1 is responsible for the defect in vacuolar fusion preventing cells from forming large vacuoles under conditions that impose vacuolar fusion (Fig. 5).

### Deletion of *FLC1* has a negative impact on the process of autophagy.

Previous studies suggested that the Flc1 homologue in *C. deneoformans* is essential for growth in low nutrient media (23). Our data suggest that Flc1 regulates vacuolar fusion under stress conditions. Autophagy is a catabolic process induced by diminishment of nutrient availability that requires vacuolar fusion mediated by the TOR pathway (39). We hypothesized that C. neoformans Flc1 plays a role in the starvation response by regulating TOR-mediated vacuolar fusion and autophagy. To test this possibility, cells were incubated on synthetic yeast nitrogen base (YNB) media without amino acids and ammonium sulfate. Consistent with our hypothesis, cells lacking Flc1 grew poorly on this low nutrient medium (Fig. 5E).

Autophagy requires mobilization of complex molecular machinery, including activation of autophagy related genes (ATG). In our study, we focused on the ubiquitin-like protein Atg8, which plays an important role in autophagosome maturation in yeast cells (40, 41). The recruitment of Atg8 is one of the triggers of vesicle expansion, and its concentration on the membrane correlates with vesicle size (42). To analyze the impact of *FLC1* deletion on autophagy, we expressed mCherry-Atg8 in the *flc1Δ* mutant. Deletion of *FLC1* did not result in mis-localization of mCherry-Atg8 on small vacuoles, but rather inhibited vacuolar fusion preventing formation of the autophagosomes (Fig. 5F).

Vacuolar fusion requires the physical presence and activation of the vacuolar H^+^-ATPase membrane sector and one of the major regulators of V-ATPase assembly is TORC1-dependent Sch9 kinase (43, 44). The process of vacuolar fusion and fission is altered by changes in pH (45, 46). To test the potential sensitivity of the *flc1*Δ cells to changes in the pH, we performed a spot growth assay on YPD media with different pH levels (pH 5, pH 7, pH 8) and incubated cells for 3 days at 24°C and 37°C. Deletion of either *FLC1* or *SCH9* did not have a negative impact on cell growth on media with acidic or basic pH (data not shown).

As our data suggested a role for Flc1 in vacuolar fusion, we predicted that Flc1 localizes to the vacuoles. However, prediction of subcellular localization using Neural Networks algorithm within DeepLoc−1.0 software suggested that Flc1 localizes to the ER. To examine the localization of Flc1, we created a fusion protein of Flc1 tagged with mCherry at the C-terminus and expressed from the endogenous Flc1 promoter, which was introduced to the *flc1Δ* strain. This Flc1-mCherry chimera was functional (Fig. 2, 3, and 5). Consistent with a potential role in vacuolar fusion, the mCherry-tagged Flc1 localized to the vacuoles (Fig. 5G).

### Deletion of *FLC1* has no effect on melanization but significantly reduces capsule size and fungal virulence in an invertebrate infection model.

**FIG 6:**
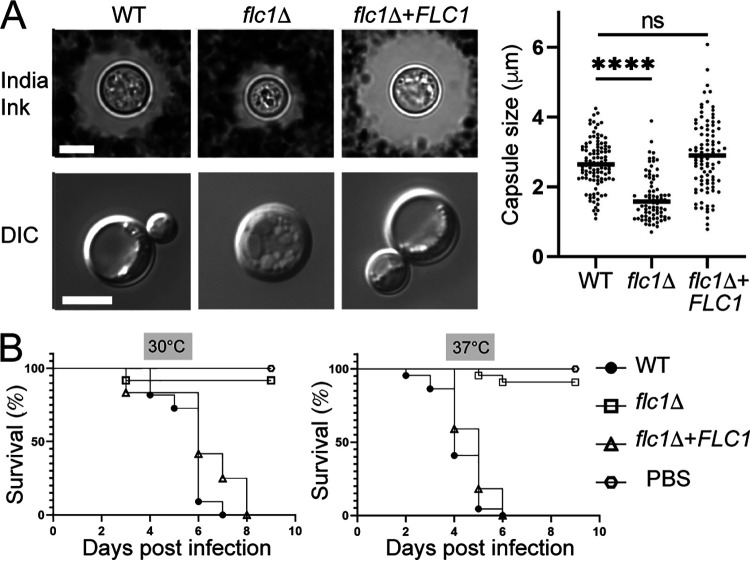
Flc1 is important for pathogenicity of C. neoformans. (A) To induce capsule formation, cells were grown for 72 h in DMEM media with 10% FBS at 37°C with 5% CO_2_. Capsules were visualized with India ink. DIC images of cells incubated in capsule inducing media indicate reduced vacuolar formation in *flc1*Δ mutant cells. Scale bar represents 5 μm. Average capsule size of *flc1*Δ mutant cells (shown in the graph to the right) differed significantly from that of the positive control (*n* > 50, *P* < 0.001, t-test). (B) Flc1 is essential for pathogenicity in the heterologous host infection model, Galleria mellonella. Each larva was injected with 10^5^ cells. Infected larvae were separated into two groups and incubated at 30°C or 37°C. In both groups survival curve for larvae inoculated with *flc1*Δ mutant differed significantly from that of the positive control (*P* < 0.0001, Mantel-Cox test; *P* < 0.0001, Gehan-Breslow-Wilcoxon test).

Deletion of *FLC1* in *C. deneoformans* suppressed production of two major virulence factors (23): (i) the antiphagocytic and macrophage-inhibiting polysaccharide capsule (47) and (ii) anti-oxidant melanin, resulting from enzymatic activity of laccase (48, 49). In contrast with *C. deneoformans*, *FLC1*-deletion in C. neoformans did not affect melanin production/laccase activity while growing on melanization agar (Fig. S1). However, we detected a major defect in capsule formation in *flc1*Δ cells in capsule-inducing conditions: Dulbecco’s modified Eagle’s medium (DMEM) with 10% fetal bovine serum (FBS) at 37°C with 5% CO_2_ (Fig. 6A). Compared with the WT and complemented strains, both the capsule and intracellular vacuole sizes were significantly reduced in *flc1*Δ cells (Fig. 6A). Supporting this observation, prior comparative transcriptional analyses indicated that the C. neoformans *FLC1* gene displays ~1.7-fold induction at 37°C with 5% CO_2_ compared with growth at 30°C (50).

10.1128/mbio.02253-22.1 FIG S1 (A, B) Cells lacking Flc1 do not proliferate in the presence of 1 M NaCl at 37°C. (A) Images representing the lowest dilution spot of cells from (B) were taken after 72 h of incubation at 37°C. The edge of the spot is at the bottom of each image. Scale bar represents 50 microns. (B) Ten-fold serial dilutions of cells were spotted onto YPD media supplemented with 1 M NaCl and incubated at either room temperature (~25°C, RT) or at 37°C (also shown as microscopy images in (A)). (C) Flc1 is not essential for melanin production. Ten-fold serial dilutions of the same strains as in (B) were spotted onto L-DOPA media. Download FIG S1, TIF file, 0.9 MB. Copyright © 2022 Stempinski et al. 2022 Stempinski et al. https://creativecommons.org/licenses/by/4.0/ This content is distributed under the terms of the Creative Commons Attribution 4.0 International license.

The absence or reduced capsule often results in diminished C. neoformans resistance to the innate immune defenses present in both invertebrate and vertebrate hosts. To assess the impact of Flc1 on virulence, we initially tested the pathogenicity of the *flc1*Δ mutant in the Galleria mellonella insect infection model, a host with innate immunity but no adaptive immune mechanisms (51). The virulence of the WT, *flc1*Δ, and the *flc1*Δ+*FLC1* complemented strains was tested at 30°C and 37°C. Inoculation of the larvae with the WT and the complemented strain resulted in death of all tested larvae within 9 and 6 days for groups incubated at 30°C and 37°C, respectively (Fig. 6B). In contrast, most of the larvae inoculated with the *flc1*Δ strain survived until the end of experiment, suggesting that Flc1 is essential for cryptococcal virulence in a Galleria mellonella infection model (Fig. 6B).

### Flc1 is essential for virulence of C. neoformans in a pulmonary mouse model of infection.

**FIG 7:**
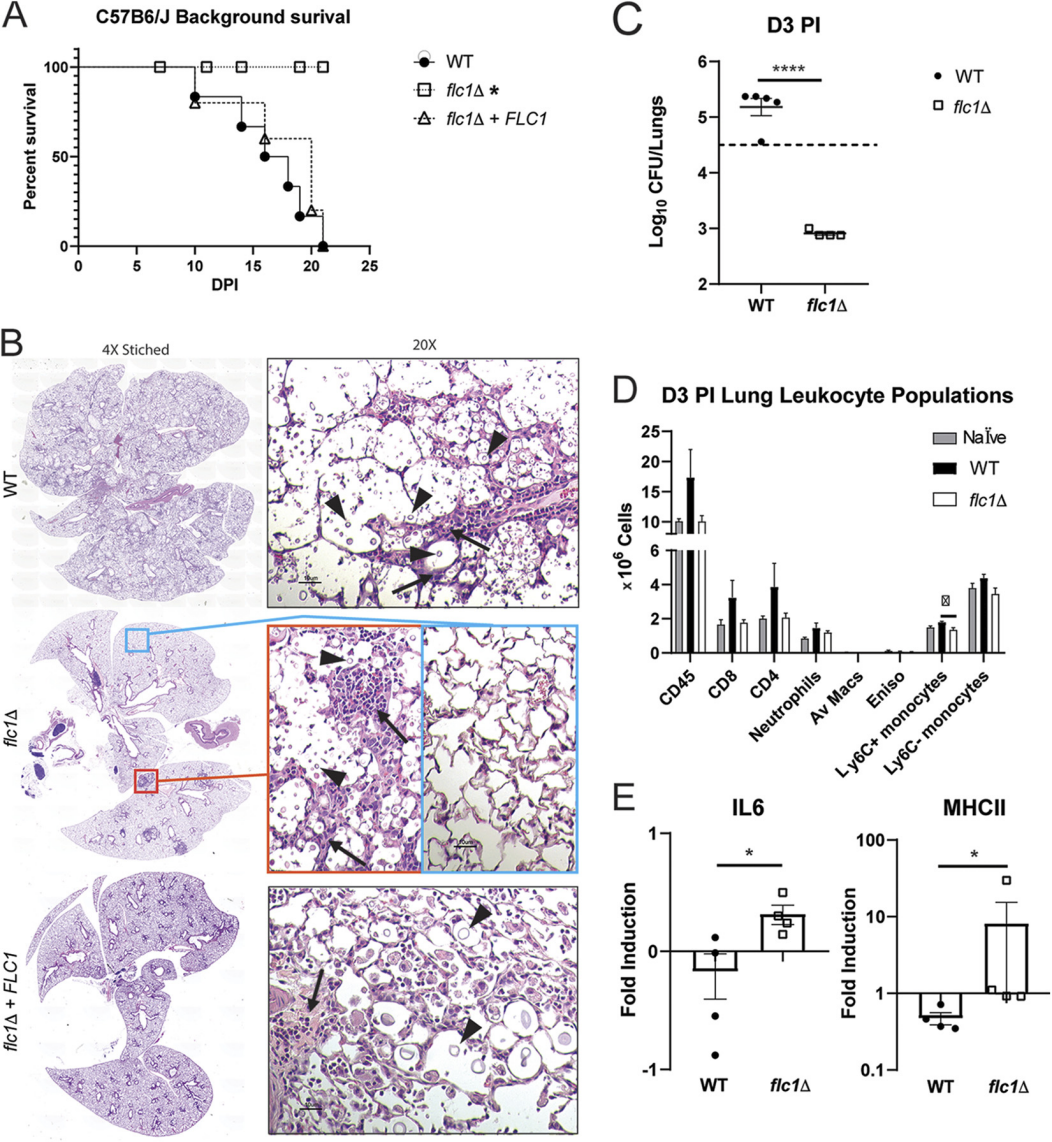
Flc1 is required for virulence of C. neoformans in a murine pulmonary model of infection. C57BL/6 male and female mice were infected with 10^5^ CFU of C. neoformans (H99 [WT], *flc1*Δ, or *flc1*Δ*+FLC1*) via intratracheal pulmonary inoculation. (A) Mice were observed for >21 days for survival analysis. Survival data are from one experiment: H99 infected (*n* = 6 mice), *flc1*Δ infected (*n* = 5 mice), or *flc1*Δ*+FLC1* (*n* = 5 mice). The data were analyzed using the Mantel-Cox test. (B) Histological images of infected C57BL/6 mice. Mice were infected as in (A) and euthanized at 20% weight loss. Following cardiac perfusion, lungs were inflated with formalin, harvested, further formalin fixed, processed for tissue blocks, cut and stained with hematoxylin and eosin (H&E). Representative whole lung histology images are “stitched” from series of 4× images (left), while the ×20 magnifications zoom on representative areas (right). On high magnification, cryptococcal cells (black triangles/arrowheads) and immune infiltrates (black arrows) are indicated. For the *flc1*Δ infected mice, cryptococci are confined to a few isolated inflammatory foci with a representative cryptococcal cluster (orange rectangle) and representative intact lungs (blue rectangle) shown on high power magnification on the right. BALB/c female mice were also infected with 10^5^ CFU of C. neoformans (H99 [WT] or *flc1*Δ) via intratracheal pulmonary inoculation. (C) Fungal burdens were evaluated in the lungs (*n* = 4 to 5 mice per group [m/g]) of mice at 3 days postinfection and analyzed by an unpaired Student's *t* test (two-tailed test). Inoculum is indicated by the dotted line. (D) Total numbers of CD45^+^ leukocytes populations were determined by flow cytometry analysis of cell isolates from enzymatically dispersed lungs at 3 days postinfection H99 [WT] or *flc1*Δ C. neoformans) or naive mice ((*n* = 4 to 5 m/g). CD45^+^ subpopulations (see Materials and Methods) were gated to calculate absolute numbers of CD4^+^ T cells, CD8^+^ T cells, neutrophils, alveolar macrophages, eosinophils, Ly6C+ monocytes, and Ly6C-monocytes. Data shown are the mean ± SEM analyzed by one-way ANOVA with Newman-Keuls multiple comparison. (E) mRNA expression levels analyzed by RT-qPCR of adherence-enriched lung leukocytes for IL-6 (left) and total lung leukocytes (right) for MHCII. Bar graphs represent the mean fold induction ± SEM from four mice per infection and analyzed for statistical significance by an unpaired Student's *t* test (two-tailed test) nonparametric Mann-Whitney. Data are from one experiment. Bars represent the means ± standard errors of the means (SEM). Significant differences are marked by asterisks: (*, *P* ≤ 0.05; ****, *P* ≤ 0.0001).

To establish the role of Flc1 in the mammalian host, we used a mouse model of primary pulmonary infection, suitable for virulence assessment (8, 52). C57Bl6/J mice were inoculated with the WT, *flc1*Δ, or *flc1*Δ*+FLC1* strains, and mouse survival was evaluated (Fig. 7A). Both WT and *flc1*Δ*+FLC1*-infected mice showed onset of mortality at 10 days postinfection (dpi), with 100% mortality seen by 21 dpi while the *flc1*Δ-infected mice showed no mortality during the studied time course. To determine how *flc1*-deletion affected the development of lung infection and pathology, histopathological analyses were conducted at the onset of mortality (10 dpi). Profound differences were found in gross pathological appearance of lungs infected with WT or *flc1*Δ*+FLC1* showing widespread cryptococcal lesions versus those with the *flc1*Δ strain that appeared healthy. Histologically, both the lungs of WT- and *flc1*Δ*+FLC1* complemented strain-infected mice showed the widespread presence of cryptococcal cells, as well as inflammatory cell infiltrates that filled the alveoli virtually across the entire lung area (Fig. 7B). Mice infected with the *flc1*Δ strain showed mostly intact lungs with a few fungal cells confined in a few isolated regions of chronic inflammation. Because these divergent phenotypes in the presence or absence of Flc1 developed in a very short time (less than 10 dpi), we investigated the role of Flc1 during the initial stage of infection. We conducted parallel intratracheal infections with the WT and *flc1*Δ strains in BALB/cJ mice and evaluated fungal burdens in the lungs at 3 dpi (Fig. 7C). We recorded a significant (~2 log) decrease in lung fungal burden of *flc1*Δ-infected mice below the level of inoculum, indicating that Flc1 is required for the initial fungal survival in mice. We next asked if the decreased survival of the *flc1*Δ strain in mice was associated with an exaggerated early/innate immune response. Leukocyte populations in the lungs analyzed by flow cytometry at 3 dpi did not show any major differences in the WT or *flc1*Δ stain infected mice, remaining near the level of naive/uninfected mice (Fig. 7D), with no evidence of increased inflammation in *flc1*Δ-infected group. We also investigated the expression profile of early immune response genes (see Materials and Methods) of both adherence-enriched leukocytes and total leukocytes by RT-qPCR (Fig. 7E). While the transcription levels of most genes were not significantly different in either group, we did observe that in the *flc1*Δ-infected mice, macrophage inflammatory cytokine IL-6 (adherence-enriched lung leukocytes) or antigen-presenting cell activation marker MHCII (total lung leukocytes) were elevated compared with WT-infected mice. Thus, even with the lower fungal burdens, the *flc1*Δ strain appeared to generate stronger macrophage/DC activation in the infected mice.

### Flc1 is essential for survival of C. neoformans cells in macrophages.

**FIG 8:**
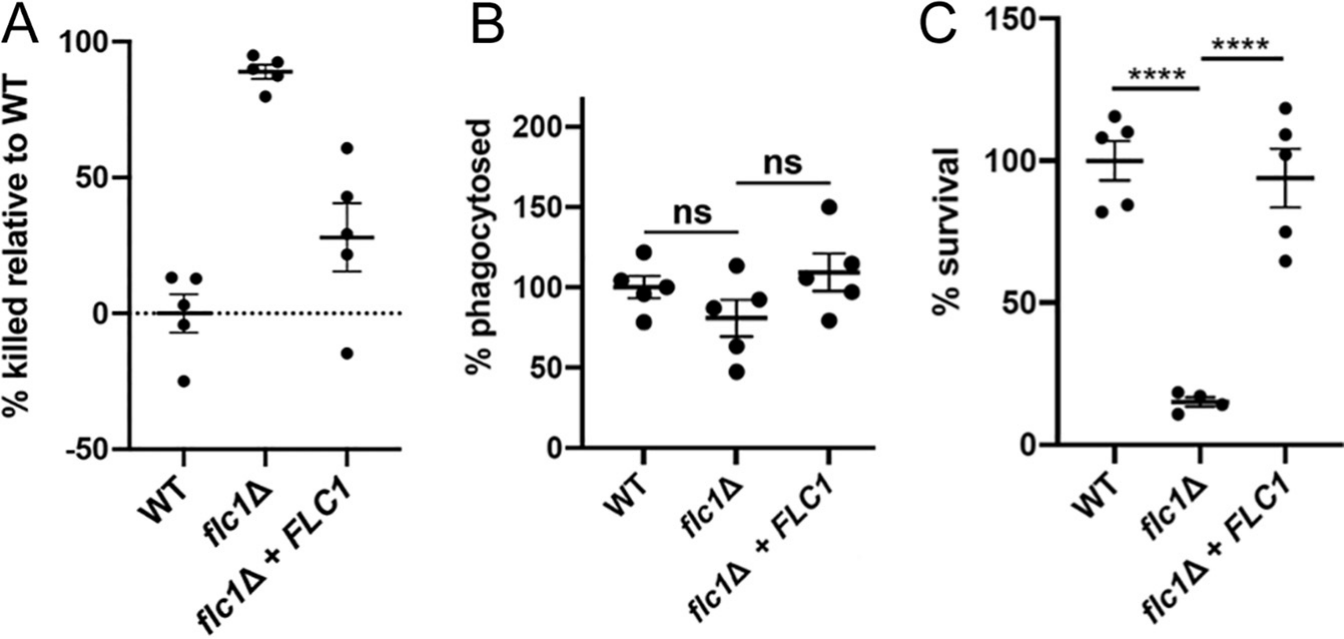
Flc1 is essential for survival of C. neoformans cells in the macrophages. The WT, *flc1*Δ mutant, and *flc1*Δ+*FLC1* complemented strains were cocultured with J774A.1 macrophage-like cells (A, B) or in the same media but without the macrophage-like cells (C). (A) The WT (*P* < 0.0001) and the complemented strain (*P* < 0.0001) were killed at a significantly lower rate than the *flc1*Δ mutant, while the killing rate was not significantly different between the WT and the complemented strain (*P* = 0.3). (B) There was no significant difference between phagocytosis rates among the strains (WT versus *flc1*Δ mutant: *P* = 0.6; *flc1*Δ + FLC1versus *flc1*Δ: *P* = 0.4; WT versus *flc1*Δ+FLC1: *P* = 0.97). (C) The WT (*P* < 0.0001) and the complemented strain (*P* < 0.0001) survived in the media without the macrophages at a significantly higher rate than the *flc1*Δ mutant, while the survival rate was not significantly different between the WT and the complemented strain (*P* = 0.8).

Given that Flc1 deletion resulted in a substantial reduction in cryptococcal burden without additional leukocyte recruitment, we tested if Flc1 was essential for fungal survival *in vitro* in macrophages, the major resident immune cell in the lungs. The WT, *flc1*Δ mutant, and the *flc1*Δ+*FLC1* complemented strains were cocultured with the J774A.1 murine macrophage-like cell line. After 24 h of coincubation with J774A.1 cells, the WT and the complemented strain displayed a similar degree of survival in coculture. In contrast, the *flc1*Δ strain was highly attenuated for survival under these conditions (Fig. 8A). All strains displayed a similar phagocytosis rate by the J774A.1 cells, indicating the difference in viable cell recovery after coculture cannot be explained by altered levels of initial phagocytosis by the J774A.1 cells (Fig. 8B). These strains were also incubated in the same growth medium in the absence of the J774A.1 cells (Fig. 8C). Few *flc1*Δ mutant cells were recovered when cultivated in the absence of J774A.1 cells, indicating that *flc1*Δ mutant cells have decreased growth fitness in the host-like conditions used in this assay, and that phagocytosis by macrophage-like cells does not provide protection against these conditions.

## DISCUSSION

Our study provides strong evidence that the Flc1 homologue is essential for pathogenicity of C. neoformans, demonstrating significantly reduced survival and/or proliferation of the *flc1*Δ mutant in a murine and insect model of cryptococcosis. The *in vitro* experiments demonstrate that the *flc1*Δ mutant exhibits severe defects in processes essential for pathogenicity, including growth under low nutrients, assembly of carbohydrate capsule, and thermotolerance when exposed to high calcium, cell wall, and hyperosmotic stresses. The importance of Flc1 in the pathogenicity of C. neoformans demonstrated here is consistent with data from a recent *C. deneoformans* study, which implicated Flc1 in pathogenicity of this closely related species (23). Importantly, genes encoding TRP-containing proteins, including Flc1 homologue in C. neoformans, were placed among 17 yeast genes with homologs in other fungal genomes, but without close homologs in other organisms (16). Thus, Flc1 may constitute a promising broad-spectrum antifungal drug target whose effective inhibition may be associated with minimal side effects.

What are the physiological roles of Flc1? Cells lacking Flc1 proliferate at 37°C suggesting that Flc1 is not required for growth at host temperature. Data from another study indicated that expression of cryptococcal *FLC1* was minimally decreased in cells exposed to oxidative stress, further suggesting that Flc1 does not act in general stress response (53). Our data provide compelling evidence that deletion of *FLC1* leads to a calcineurin-dependent increase in nuclear accumulation of Crz1 and suggest that this is due to aberrantly increased cytosolic calcium. While this phenotype may be an indirect compensatory effect of the loss of *FLC1*, the involvement of FLC homologues in calcium homeostasis has been previously reported in other fungal species (18, 21, 22, 24). Our data suggest Flc1 localizes to the vacuole, which could be a location for its calcium transport activity. Flc1 could act there as either a calcium sensor or directly as a calcium channel. Similar to other fungi, the C. neoformans vacuole is a major calcium storage organelle, and two calcium transporters, Vcx1 and Pmc1, play overlapping roles in regulating cytosolic calcium and are responsible for tolerance to external calcium levels (25, 54). The *vcx1*Δ *pmc1*Δ cells exhibit a significant increase in cytosolic calcium levels, and Pmc1 is essential for both the progression of pulmonary infection and brain colonization in mice (54).

Our data indicate the elevated calcineurin signaling in the C. neoformans *flc1*Δ mutant leads to aberrant cell wall composition with overaccumulated chitin, a defect that may explain observed hypersensitivity to cell wall damaging agents at 37°C and which is consistent with cell wall integrity defects observed in *C. deneoformans flc1*Δ mutant and homologous mutants in other fungi (17, 18, 21, 23). In addition, C. neoformans mutant lacking Flc1 that was recently identified in a genetic screen, has been shown to exhibit a partial cell separation defect at 37°C, consistent with the aberrant chitin distribution defect reported here (55).

Interestingly, in contrast to several known cell wall integrity mutants (56), exposure of *flc1*Δ cells to 1 M sorbitol did not rescue cell growth but rather led to even more pronounced chitin accumulation and prevented cell growth at 37°C. This finding contrasts with data from C. albicans where 1 M sorbitol partially rescued growth of the *flc1*Δ *flc2*Δ mutant, even though a lack of these FLC homologues in C. albicans, like *flc1*Δ in C. neoformans, leads to hypersensitivity to cell wall damaging agents and results in overaccumulation of chitin in the cell wall (17). The *flc1*Δ C. neoformans cells were also hypersensitive to high NaCl concentration specifically at 37°C (Fig. S1). These findings suggest Flc1 plays a role in osmoregulation specifically at 37°C that is not shared with its homologues in C. albicans. The direct relevance of microbial osmoregulation in the setting of infection is complex as osmolarity of mammalian cells and tissues is under strict regulation although hyperosmolarity of some cells, most notably renal tissue, occurs physiologically (57). Strikingly, the C. neoformans *flc1*Δ mutant exhibited vacuolar fragmentation similar to the class C vacuolar protein sorting (vps) mutants described in S. cerevisiae (58), whereas the WT displayed enlarged vacuoles under conditions of combined 37°C and 1 M sorbitol. The fact that exposure of the WT cells to combined hyperosmotic and high temperature stress leads to vacuolar enlargement was surprising as hyperosmotic stress alone is expected to result in an opposite effect and result in vacuolar fission (59, 60). However, a combination of these two stresses has not been investigated for its effects on vacuolar dynamics, and further mechanistic explanation of our results will require more studies. Importantly, the *flc1*Δ mutant was unable to perform other processes that are accompanied by vacuolar fusion in the WT and that are relevant to pathogenicity, namely, growth on nutrient-poor media, and formation of capsule.

**FIG 9:**
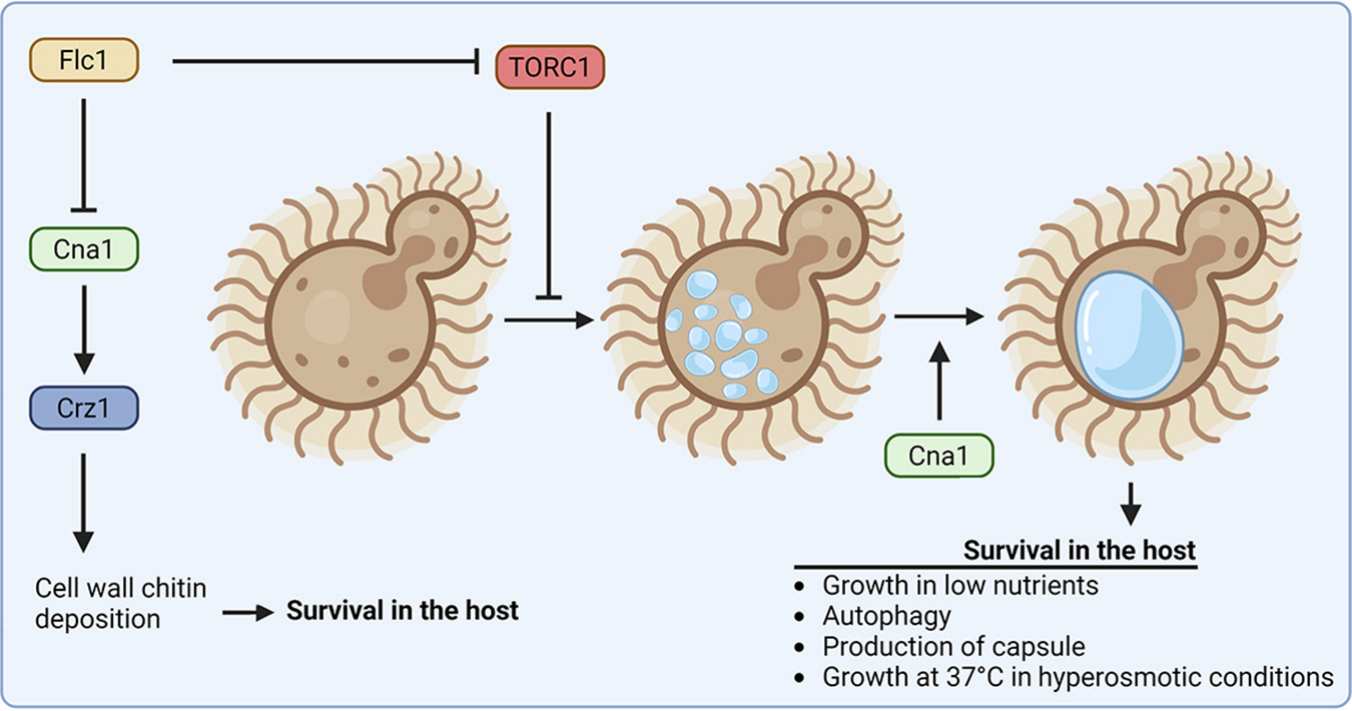
A model depicting the function of Flc1 in vacuolar biogenesis in C. neoformans. Flc1 contributes to vacuolar homeostasis via negative regulation of the TORC1 pathway. This is critical for growth in low nutrients and for the formation of capsule, both contributing to fitness in the host. In the absence of Flc1, under conditions that trigger vacuolar fusion, for instance at 37°C in the presence of 1 M sorbitol, cells contain only small and fragmented vacuoles. In addition, cells lacking Flc1 exhibit overaccumulation of cell wall chitin caused by hyperactive calcineurin (Cna1), which may additionally compromise fitness in the host. The effect of *FLC1* deletion on Cna1 signaling may reflect a direct regulation of calcium homeostasis by Flc1 or may be a compensatory consequence of the absence of Flc1. Cna1 may also act in vacuolar fusion as inhibition of Cna1 resulted in cells with small vacuoles under conditions that promote vacuolar enlargement (image created with Biorender).

Collectively, our data indicate for the first time a crucial role for the fungal TRP homologue in vacuolar fusion and provide mechanistic explanation of the requirement for Flc1 in several processes that are accompanied by this morphological change in vacuolar homeostasis as illustrated by the working model proposed in Fig. 9. Several factors, compartmentalized in distinct vacuolar membrane domains, participate in the process of vacuolar fusion, including Rab/Rho GTPases, SNAREs, and actin, as well as the regulatory lipids, phosphatidic acid, diacylglycerol, ergosterol, phosphatidylinositol phosphates, and sphingolipids (61–63). S. cerevisiae FLC mutants exhibit a severe depression of sphingolipid biosynthesis, which, if conserved in C. neoformans, may potentially explain the requirement for Flc1 in vacuolar fusion reported here (64). In addition to core fusion apparatus, multiple layers of regulatory mechanisms exist that impact vacuolar dynamics according to environmental cues (59). Most notably, a multiprotein complex known as TORC1 that contains TOR1/2 kinases is a well-established conserved regulator of vacuolar dynamics responding to nutrient supply (38, 65, 66). Our data suggest that one role of Flc1 is to inhibit TORC1 complex under certain stress conditions, as treatment with rapamycin led to a restoration of large vacuoles in the *flc1*Δ mutant at 37°C in the presence of 1 M sorbitol. A growth defect of the *flc1*Δ mutant under poor nutrient conditions is consistent with this possibility. In S. cerevisiae, expression of Flc2 is significantly increased (~2-fold change) in cells treated with TOR inhibitors (67) and the mammalian TRP channel protein TRPML1 is involved in regulation of TORC1 pathway and lysosome biogenesis (15, 68, 69). Thus, fungal FLC proteins may play a conserved role in low nutrient response via TOR pathway (Fig. 9).

Our results demonstrate that inhibition of calcineurin in the WT incubated with 1 M sorbitol at 37°C prevents vacuolar fusion. This finding implicates a role for calcineurin in vacuolar homeostasis during stress, a possibility that has not been thoroughly explored in fungal cells but is consistent with studies in mammals. For instance, lysosomal calcium release through TRPML1 activates calcineurin, which binds and dephosphorylates transcription factor TFEB, thus promoting its nuclear translocation and lysosomal fusion during autophagy (70). Our finding indicating hyperactive calcineurin in the *flc1*Δ mutant seems to contradict this possibility. However, reports on calcium microdomains within the cell suggest that the effects of local calcium release may compartmentalize distinct calcium-dependent responses (71). Thus, it is possible that deletion of *FLC1* results in activation of the calcineurin-Crz1 pathway, while inhibiting calcineurin signaling results in vacuolar fragmentation under stress-specific conditions.

Our results demonstrate a requirement for Flc1 in virulence. Impaired survival of the *flc1*Δ mutant in the host can likely be explained by our observation of reduced fitness in host-like growth conditions. However, this strain also demonstrates a reduction of capsule that regulates microbe-host interactions. Together, our data suggest that Flc1 contributes to the overall fitness of C. neoformans in the presence of stress, as well as the appropriate expression of fungal factors that promote microbe survival during host cell interaction. Accordingly, the *flc1Δ* strain is unable to survive in either a murine model of infection or in an insect model that lacks adaptive immunity. Furthermore, we see 1,000-fold suppression of fungal burden at 3 dpi in mice, accompanied by hallmarks of stronger macrophage/DC activation in the *flc1*Δ-mutant-infected mice compared with WT, despite the fact that these changes occur prior to nonresident leukocyte recruitment into the lung, and in the absence of fungal expansion we see in the WT strain. While future studies are needed to fully understand how Flc1 alters fungal interactions with the mammalian immune defenses, our data collectively point out that Flc1 is required for both cryptococcal fitness and its resistance to the innate mechanisms.

In summary, we found that Flc1 plays a crucial role in C. neoformans stress responses and virulence, which is linked to its role in Ca^2+^ signaling/homeostasis. Deletion of Flc1 results in abnormal distribution of chitin, inhibition of the vacuolar fusion, and interruption of autophagy in C. neoformans. Importantly, cells lacking *FLC1* demonstrate a significant loss of virulence in both invertebrate and mammalian infection models, identifying Flc1 protein as a promising potential drug target.

## MATERIALS AND METHODS

### Strains and media.

WT C. neoformans (formerly var. *grubii*) strain H99 (MAT α) and other strains congenic to H99 (listed in Table 1) were used in this study. The original C. neoformans *flc1*Δ mutant strain was generated by Hiten Madhani’s group at the University of California San Francisco (UCSF) and purchased from the Fungal Genetics Stock Center (FGSC). An independent *flc1*Δ mutant strain in the H99 strain background was generated by biolistic transformation combined with insertion of the NEO cassette by homologous recombination in the H99 strain (72). The Flc1-mCherry was created in *flc1*Δ background by biolistic transformation of modified pLK25 plasmid (pPS2), which resulted in fusing the mCherry protein at the C-terminus of Flc1, as detailed below. All strains were routinely cultured on YPD medium (1% yeast extract, 2% peptone, 2% agar, and 2% dextrose).

**TABLE 1:** List of strains used in this study

Strain	Origin	Genotype	Ref.
H99	N/A	WT	87
LK141	KN99**a**	*CDC3-mCherry:NEO*	33
LK174	KN99**a**	*cdc12::NEO*	33
LK310a	KN99**a**	*mCherry-ATG8:HYG*	This study^*a*^
LK325	KN99**a**	*crz1::NAT CRZ1-mCherry:NEO*	This study^*a*^
EA85	KN99**a**	*BUD4-mCherry:NEO*	This study^*a*^
PS43	H99 α	*flc1::NAT*	This study
PS44	H99 α	*flc1::NAT FLC1-mCherry:NEO*	This study
PS46	PS43xLK325	*flc1::NAT CRZ1-mCherry:NEO*	This study
PS50	PS43xE85	*flc1::NAT BUD4-mCherry:NEO*	This study
PS52	PS43xLK174	*flc1::NAT cdc12::NEO*	This study
PS57	PS43xLK310a	*flc1::NAT mCherry-ATG8:HYG*	This study
PS61	PS43xLK141	*flc1::NAT CDC3-mCherry:NEO*	This study

a Strains LK310a, LK325, and EA85 were generated in Heitman Lab (Duke University). LK310 was made by transforming strain KN99**a** with an LKB55-based plasmid that expresses Atg8-encoding sequence from *GPD1* promoter (88). LK325 was made by transforming strain YPH271 (*CRZ1::NAT*) with plasmid pXW15, which encodes Crz1-mCherry (26). EA85 was generated by replacing the STOP codon in a gene encoding a Bud4 homologue in the KN99**a** strain with the sequence encoding mCherry based on plasmid LKB25 (33) (EA85 is included in a manuscript that is being currently submitted for publication by Kozubowski Lab).

### Disruption and complementation of *FLC1* in C. neoformans.

The C. neoformans *FLC1* gene replacement sequence, flanked by 1Kb of the 5’ untranslated region (5’UTR) and 1Kb of the 3'UTR regions, was amplified by PCR from the genomic DNA of the *flc1*Δ mutant from the Madhani collection with the set of primers W1 5′-CTAGTCGAGTGGTGACGCCGTT-3′ and W6 5′-ACCGACGATCAAGCGGTATTTG-3′. The PCR product was introduced into WT H99α strain by biolistic transformation (72). The transformed cells were selected on YPD media containing 100 μg/mL nourseothricin.

To create a complemented strain (*flc1*Δ+*FLC1*), the genomic region encoding Flc1 along with 1,494 bp of the 5′-UTR region was amplified with the set of primers LUKO_477 5′-CTATCTAGACGTGTCAAGATGATGTTGTC-3′ and LUKO_478 5′-TACTCTAGAATACCGTCTTGACCATTGC-3′ with introduced XbaI cutting sites at both ends and inserted into the XbaI-digested vector pLKB25 (33), resulting in a plasmid (pPS1), which expresses the Flc1 fused with mCherry at the C-terminus. The cloned pPS1 vector was then introduced into the *flc1*Δ mutant strain by biolistic transformation (72). The transformed cells were selected on YPD media containing 200 μg/mL Geneticin.

### Microscopy.

For microscopy, cells were routinely cultured in YPD medium overnight at RT (~25°C, unless specified otherwise), diluted 1:10, and then incubated for an additional 3 h.

For cell wall imaging, 1.5 mg/mL of CFW was added to the cells for 10 min followed by washing with phosphate buffered saline (PBS). For testing the effect of host temperature, cells were incubated 4 h in YPD at 37°C. Cells were observed under the Leica DMi8 inverted fluorescence microscope with a 100× objective. Images were analyzed using Leica Application Suite X (LAS X) software. Cell size was quantified using ImageJ software (NIH).

To estimate nuclear translocation of Crz1-mCherry, overnight cultures were refreshed in 2 mL of YPD and incubated for an additional 2 h at ~25°C (RT). Cells were photographed under the Zeiss Axiovert 200M inverted fluorescence microscope with the 100× objective. The imaging was repeated after the cells were incubated for 4 h at 37°C. Subsequently, cell cultures were transferred back to RT and incubated for 15 and 60 min before subsequent microscopy imaging. Nuclear enrichment of Crz1-mCherry was established as a ratio of the intensity of the nuclear fluorescent signal to the intensity of the cytoplasmic fluorescent signal of Crz1-mCherry using ImageJ software (NIH). Each observation was performed three times and at least 80 cells were scored.

### Intracellular calcium measurement.

To estimate intracellular calcium level, overnight cultures were refreshed in YPD media and incubated for 2 h at RT or 37°C. Subsequently, cells were washed twice with PBS and stained with 5 μM calcium indicator Fluo-3 AM (Invitrogen) in the dark, maintaining the respective temperatures (RT or 37°C) for 30 min. After staining, cells were washed with PBS and the calcium levels were measured by fluorescence flow cytometry with CytoFLEX (Beckman) using a parameter of the excitation to emission wavelengths (485 nm/530 nm).

### Serial spot dilution assay.

Overnight cultures were refreshed in 2 mL of YPD and incubated for an additional 2 h at RT. Next, cells were washed twice with PBS, adjusted to 3.3× 10^4^ cells/mL and used for a 10-fold serial dilution (10^0^ to 10^−3^). Cells were spotted onto semisolid media in 3 μL spots and the plates were imaged after 2 to 4 days.

### Cryptococcus neoformans mating.

The *flc1*Δ strain was mated with the following strains: LK174 (*cdc12*Δ), LK141 (Cdc3-mCherry), EA85 (Bud4-mCherry), and LK310a (mCherry-Atg8). To induce mating, cultures of two different mating types (MATα and MATa) were grown overnight in YPD liquid at RT, washed with PBS, mixed in 1:1 ratio, and spotted onto Murashige Skoog (MS) mating media (73). Mating mixtures were incubated in the dark at RT for 10 to 12 days, as described (73). For each strain, at least 32 spores, originating from at least four basidia, were transferred onto YPD semisolid media with the use of SporePlay Tetrad Dissection Microscope (Singer Instruments). To confirm results of the mating, strains were tested for growth on YPD media supplemented with an appropriate antibiotic.

### Vacuolar size measurement.

After overnight incubation at RT in YPD liquid media, cells were refreshed and transferred into YPD media supplemented with 1 M sorbitol and incubated for 4 h at 37°C. To inhibit calcineurin or TOR pathway, media were supplemented with cyclosporine A (100 μg/mL) or rapamycin (1 μg/mL), respectively. For vacuolar and nuclear imaging, during the last 20 min of incubation, cell cultures were labeled with FM4-64 (1 μg/mL) and Hoechst dye (10 μg/mL), respectively. After the incubation, cells were washed with PBS and observed under the Leica DMi8 inverted fluorescence microscope with a 100× objective. The average size of the vacuoles in the tested population was established by measuring the diameter of the biggest vacuole in the cell using ImageJ software. Measurements were performed for at least 50 cells in each population.

### Melanin production assay.

Overnight cultures were refreshed in 2 mL of YPD and cultured for an additional 2 h. Next, cells were washed twice with PBS, adjusted to 3.3× 10^4^ cells/mL and used for a 10-fold serial dilution (10^0^ to 10^−3^). Cells were spotted onto semisolid l-3,4-dihydroxyphenylalanine (L-DOPA) media (15 mM glucose, 13 mM glycine, 10 mM MgSO_4_, 29.4 mM KH_2_PO_4_, 3 mM thiamine, 2% Agar, 1 mM L-DOPA) in 3 μL spots and cultured at RT for 3 days prior to imaging (74).

### Capsule formation assay.

Capsule was induced based on the original protocol described by Granger D.L. et al. (75, 76). Overnight cultures were washed twice with PBS, resuspended in the DMEM culture media (Corning, Corning, NY) with addition of 10% FBS (Neuromics, Edina, MN), and transferred to 6-well plates with 10^6^ cells in each well. Cells were incubated for 5 days at 37°C with 5% CO_2_, stained with India ink, and then observed under the Leica DMi8 inverted fluorescence microscope with a 100× objective. The size of the capsule was quantified using ImageJ software by measuring the width of the halo created by the capsule, which is visualized by negative staining with India ink.

### Autophagy and vacuolar fusion assay.

To visualize changes in the localization of Atg8 in the *flc1*Δ mutant strain, we created *flc1*Δ mCherry-Atg8 strain by mating the *flc1*Δ mutant strain with LK310a (mCherry-Atg8). After overnight incubation in YPD media at RT, strains were washed in PBS and cultured in 2 mL of YPD or YNB without amino acids and without ammonium sulfate at 30 or 37°C for 4 h. The subcellular localization of mCherry-Atg8 was observed under the Leica DMi8 inverted fluorescence microscope with a 100× objective.

### Galleria mellonella virulence assay.

The assay was performed based on a procedure established for studies involving C. neoformans (77). Galleria mellonella larvae (250 to 300 mg in weight) were selected and used for the following experimental groups (12 larvae per group) according to the inoculum: PBS as a negative control, WT stain H99, *flc1*Δ mutant strain, and the complemented strain *flc1*Δ*+FLC1*. Prior to the infection, C. neoformans strains were grown overnight in YPD media at RT, refreshed for 2 h and adjusted to 2 × 10^4^ CFU/μL in PBS. Each larva was injected with 5 μL of the inoculum into the last right proto-leg using a 0.5 mL syringe. After inoculation, larvae were kept in separate Petri dishes and incubated in darkness at 30°C or 37°C. The viability of the larvae was assessed daily based on change of color and response to touch.

### Virulence study based on murine model of infection.

**(i) Mice.** C57BL/6J (stock #:000664) background mice from Jackson Laboratory and bred at the Ann Arbor Veterans Affairs Medical Center and BALB/cJ (stock #:000651) mice were purchased from Jackson Laboratory (Bar Harbor, ME) and housed under specific-pathogen-free conditions at the Ann Arbor Veterans Affairs Medical Center. Female BALB/cJ mice were 8 to 10 weeks old at the time of infection while male and female C57BL/6J mice were 17 to 25 weeks old at the time of infection. Mice were humanly euthanized via CO_2_ inhalation at the selected time points postinfection. All experiments were approved by the Veterans Affairs Institutional Animal Care and Use Committee (protocol 1408-004) and were performed in accordance with NIH guidelines and the Guide for the Care and Use of Laboratory Animals. For mortality studies, animals were euthanized when they lost 20% body weight.

**(ii) C. neoformans infection.** The WT, *flc1*Δ, and *flc1*Δ+*FLC1* strains were grown for 4 days in Sabouraud dextrose broth (Difco) at 34°C, then cells were washed in PBS, counted on a hemocytometer with trypan blue, and adjusted to a concentration of 3.3 × 10^6^/mL prior to the infection. Mice were anesthetized via i.p. injection with ketamine/xylazine (100/6.8 mg/kg body weight) prior to intratracheal infection procedure (52, 78), receiving 10^5^ organisms in 30 μL PBS. Serial dilutions of the inocula were plated on Sabouraud dextrose agar (SDA) plates to confirm the CFU number of each inoculum.

**(iii) Lung leukocyte isolation.** Mice were euthanized with CO_2_ followed by PBS perfusion. The lungs were aseptically removed, transferred to sterile GentleMACs C tubes (Miltenyi), and processed using enzymatic and mechanical dispersion protocol described previously (79). Small aliquots of the dispersed lung prep were then collected and processed for organ fungal burden measurements while the remainder was processed following established protocol (80) in order to obtain a single cell suspension highly enriched in lung leukocytes. Isolated cells were vaunted and analyzed by flow cytometry or for gene expression (following RNA extraction) in the total cell prep, and the adherence-enriched macrophage prep.

**(iv) Flow cytometry.** All isolated cells were stained with fixable live/dead dye (Life Technologies), blocked with anti-CD16/32, and stained with CD45. Flow cytometry protocol was adapted from previous work (81, 82). Gating of CD45 subpopulations on CD4 and CD8 generated CD4^+^ T cells, CD8^+^ T cells, CD4^+^/CD8^+^ cells and CD4^−^/CD8^−^ cells. The CD4^−^/CD8^−^ population was further divided into neutrophils (CD11b^+^/Ly6G^+^), alveolar macrophages (Ly6G^−^/SiglecF^+^/CD11c^+^), eosinophils (Ly6G^−^/SiglecF^+^/CD11C^−^), Ly6C^+^ monocytes (Ly6G^−^/SiglecF^−^/Ly6C^+^) and Ly6C^−^ monocytes (Ly6G^−^/SiglecF^−^/Ly6C^−^). All antibodies were purchased from BioLegend. Clone numbers for antibodies used in this study: CD45 (30-F11), CD4 (GK1.5), CD8 (53-6.7), CD11b (M1/70), CD11c (N418), Ly6C (HK1.4), Ly6G (1A8), SiglecF (E50-2440). Data were collected on an LSRII cytometer (BD) and analyzed via FlowJo (Treestar).

**(v) Fungal burdens.** To quantify fungal burdens for each organ, aliquots of organ homogenate were serially diluted in distilled water, and 10 μL aliquots were plated on SDA plates. The CFU per organ was calculated as mean from the minimum of two replicate platings.

**(vi) Histology.** Histology was performed as previously described (81). Lungs were slightly perfused with PBS, then inflated with 10% neutral buffered formalin prior to removal and fixation with 10% neutral buffered formalin. They were then paraffin embedded and cut into 5-mm sections for H&E staining by McClinchey Histology Lab (Stockbridge, MI). The sections were visualized by light microscopy with microphotographs taken by the Digital Microphotography system DFX1200 with ACT-1 software (Nikon, Tokyo, Japan) or with the BZ-X810 fluorescence microscope then analyzed with DB-X800 analyzer software (Keyence Itasca, IL) for stitched images.

**(vii) Gene expression.** Cells from the leukocyte isolation were stored in TRIzol (Life Technologies) at −80°C prior to extraction. cDNA was generated using the Quantitech reverse-transcription kit (Qiagen). Gene expression of TNF-α, IFN-γ, IL-12, IL-10, IL-4, IL-13, IL-17, CD86, CD80, MHCII, iNOS, Arg1, Ym2, and IL-6 genes was determined using a SYBR green-based detection (Radiant Green master mix; Alkali Science) on a LightCycler 96 thermocycler (Roche). Data were as fold induction normalized to 18S housekeeping mRNA.

**(viii) Statistical analysis.** Values are reported as means with variance indicated. GraphPad Prism v8 software (GraphPad Software, San Diego, CA) was used to preform statistical analyses as indicated in figure legends. *P* values were adjusted for multiple comparisons whenever more than two groups were compared with *P* < 0.05 marking statistical significance.

### Macrophage survival assay.

J774A.1 macrophage-like cells, originally derived from murine BALB/c reticulum cell sarcoma, were maintained in macrophage medium (DMEM, heat-inactivated FBS, penicillin-streptomycin [Gibco 15140-122], as well as minimal essential medium [MEM] nonessential amino acid solution [Gibco 11140-050]) in a humidified 37°C incubator with a 5% CO_2_ atmosphere, as described (83, 84). Cells were passaged to fresh macrophage medium in tissue culture flasks twice weekly to ensure good cell viability during the experiment. Survival of WT C. neoformans, *flc1*Δ mutant, and a *flc1*Δ+*FLC1* complemented strains within the macrophage-like cells was assessed by aliquoting 10^5^ viable macrophage-like cells into individual wells of a 96-well plate. The plate was incubated overnight in a 37°C incubator supplemented with 5% CO_2_. J774A.1 cells were activated with the addition of 10 nM phorbol myristate acetate (PMA) to the cells in macrophage medium and incubation at 37°C with 5% CO_2_ for 1 h. Cryptococcal cells were incubated overnight at 30°C while shaking at 150 rpm. Overnight cryptococcal cultures were washed and resuspended in macrophage medium and standardized to 10^6^ cells/mL, followed by opsonization with anti-glucuronoxylomannan (GXM) monoclonal antibody (Mab) 18B7 (1 μg/mL) for 1 h in a 37°C incubator supplemented with 5% CO_2_. Macrophage medium was removed from the J774A.1 cells in the 96-well plate and 100 μL of opsonized and standardized cryptococcal cells was added to each well. Cryptococcal cells suspended in macrophage medium were also added to wells without J774A.1 cells to assess survival of the fungal cells under these conditions in the absence of macrophage-like cells. In addition, the standardized C. neoformans cultures were plated on YPD plates for quantification of the input culture. The cocultures were incubated for 1 h in a 37°C incubator with 5% CO_2_. Following incubation, wells with cocultures were washed once with PBS to remove extracellular yeast without disrupting the monolayer of J774A.1 cells at the bottom of the wells, and fresh macrophage medium was added to each well. The macrophage-like cells were incubated at 37°C with 5% CO_2_ for 24 h. Likewise, the fungal cells added to wells without macrophage-like cells were incubated at 37°C with 5% CO_2_ for 24 h. Cryptococcal cells that survived were harvested by disrupting the J774A.1 monolayer with sterile, distilled H_2_O and plating on YPD plates for quantification of cells that survived in the macrophages and in these growth conditions. To assess the extent of phagocytosis of these cryptococcal strains by the J774A.1 cells, the same procedure as above was followed to prepare the cocultures. Following the 1 h of incubation of the coculture and washing with PBS to remove nonengulfed fungal cells, the host cells were lysed with sterile H_2_O. The previously phagocytosed cryptococcal cells were immediately harvested and plated for quantitative culture. Five biological replicates of each strain were analyzed in each assay and one-way ANOVA and Tukey’s multiple-comparisons tests were run to assess statistical significance among the survival and phagocytosis percentages.

### *In silico* sequence data analysis.

Sequences of proteins analyzed in this study were obtained from the NCBI website (www.ncbi.nlm.nih.gov). The search for potential Flc1 homologues was performed by analysis of amino acid sequence homology with Domain Enhanced Lookup Time Accelerated BLAST (DELTA-BLAST). An additional analysis of fungal proteins containing Transient Receptor Potential domain (TRP; PF06011) was performed with OrthoMCL 6.5 algorithm (https://orthomcl.org/). Protein domain analysis was performed using the Pfam database (Pfam version 34.0). Selected proteins, containing TRP domains (PF06011), representing Basidiomycota and Ascomycota, were utilized to create the phylogenetic tree. Phylogenetic analysis was performed with ngphylogeny software (ngphylogeny.fr) with standard settings, and visual presentation of the results was created with Interactive Tree Of Life iTOL v6 (https://itol.embl.de/) (85, 86). The signal peptide prediction for Flc1 was performed with the TargetP-2.0 server (89). Multiple amino acid sequence alignment using conserved domain and local sequence similarity was performed with the COBALT alignment tool (https://www.ncbi.nlm.nih.gov/tools/cobalt/re_cobalt.cgi).
