# Supplementary material for: The Cryptococcus neoformans Flc1 Homologue Controls Calcium Homeostasis and Confers Fungal Pathogenicity in the Infected Hosts
Source: mBio. 2022 Sep 28;13(5):e02253-22. doi: 10.1128/mbio.02253-22 (PMC9600462; doi:10.1128/mbio.02253-22)
Supplement: TABLE S1 [file mbio.02253-22-s0002.pdf]

Table S1 Proteins with significant homology (% identity is indicated) to *C. neoformans* Flc1
Homologues of Flc1 have been found among representative fungal species. Characteristic feature of all homologues of Flc1 is the TRP (PF06011) domain. Most of the FLC homologues from Ascomycota and Basidiomycota possess ML-like (PF14558) domain. In contrast to other analyzed species, homologues of Flc1 in phylum Mucormycota contain ML (PF02221) domain.

Species	Gene name	% Ident.	Length (aa)	Domain 1	Domain 2
**Basidiomycota**					
C. deneoformans	CNI02660	93.5%	768	ML-like (PF14558)	TRP (PF06011)
C. deneoformans	CNA00570	21.9%	1284	Absent	TRP (PF06011)
U. maydis	UMAG_10761	28.4%	814	ML-like (PF14558)	TRP (PF06011)
U. maydis	UMAG_11683	11.9%	1480	Absent	TRP (PF06011)
**Ascomycota**					
S. cerevisiae	*Flc1*	22.9%	793	ML-like (PF14558)	TRP (PF06011)
S. cerevisiae	*Flc3*	22.8%	802	ML-like (PF14558)	TRP (PF06011)
S. cerevisiae	*Flc2*	22.0%	783	ML-like (PF14558)	TRP (PF06011)
S. cerevisiae	YOR365C	20.0%	703	ML-like (PF14558)	TRP (PF06011)
S. pombe	*Pkd2*	20.7%	710	ML-like (PF14558)	TRP (PF06011)
S. pombe	*Trp663*	18.4%	687	ML-like (PF14558)	TRP (PF06011)
S. pombe	*Trp1322*	17.0%	862	Absent	TRP (PF06011)
A. fumigatus	*FlcA*	22.4%	721	ML-like (PF14558)	TRP (PF06011)
A. fumigatus	*FlcC*	24.3%	723	ML-like (PF14558)	TRP (PF06011)
A. fumigatus	*FlcB*	21.6%	612	ML-like (PF14558)	TRP (PF06011)
A. fumigatus	AFUA_7G01610	15.1%	1128	ML-like (PF14558)	TRP (PF06011)
C. albicans	*Flc2*	20.7%	811	ML-like (PF14558)	TRP (PF06011)

C. albicans	*Flc3*	19.9%	718	ML-like (PF14558)	TRP (PF06011)
C. albicans	*Flc1*	20.0%	808	ML-like (PF14558)	TRP (PF06011)
C. albicans	*Flc4*	18.0%	1093	Absent	TRP (PF06011)
N. crassa	NCU01036	20.7%	766	ML-like (PF14558)	TRP (PF06011)
N. crassa	NCU05785	23.1%	768	ML-like (PF14558)	TRP (PF06011)
N. crassa	NCU09253	22.7%	950	ML-like (PF14558)	TRP (PF06011)
N. crassa	NCU01978	21.6%	948	ML-like (PF14558)	TRP (PF06011)
N. crassa	NCU09374	16.4%	1448	ML-like (PF14558)	TRP (PF06011)
**Mucormycota**					
M. circinelloides	HMPREF1544_11895	17.5%	818	ML (PF02221)	TRP (PF06011)
M. circinelloides	HMPREF1544_10413	15.2%	697	Absent	TRP (PF06011)
