# Supplementary figures and images for: The Cryptococcus neoformans Flc1 Homologue Controls Calcium Homeostasis and Confers Fungal Pathogenicity in the Infected Hosts
Source: mBio. 2022 Sep 28;13(5):e02253-22. doi: 10.1128/mbio.02253-22 (PMC9600462; doi:10.1128/mbio.02253-22)

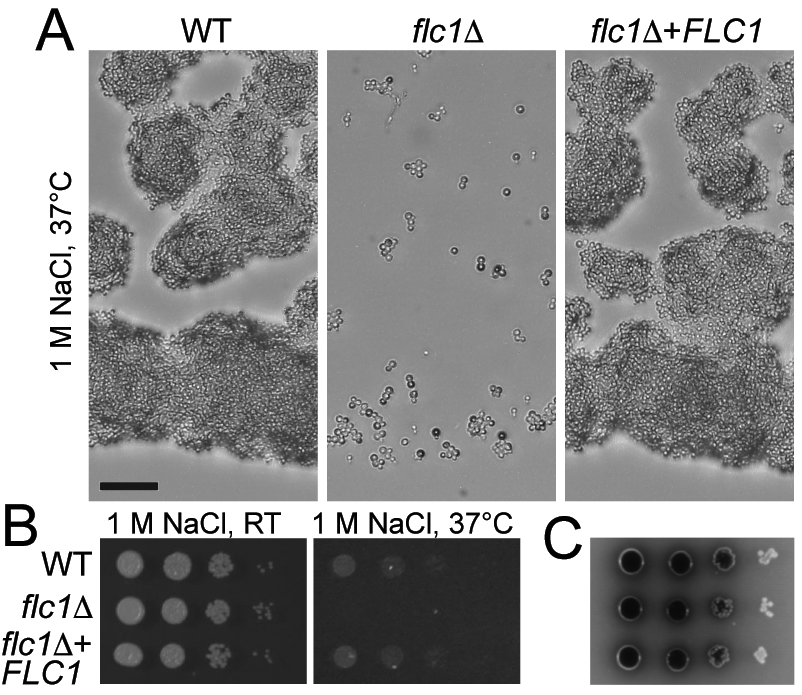

Supplement: FIG S1 [file mbio.02253-22-s0001.tif]
